# A Training Program to Support Patient Engagement in Primary Health Care Research: Co-Design, Implementation, and Evaluation Study

**DOI:** 10.2196/65485

**Published:** 2025-06-05

**Authors:** Amanda L Terry, Lorraine Bayliss, Leslie Meredith, Eugene Law, Rob Van Hoorn, Sandra Regan

**Affiliations:** 1 Centre for Studies in Family Medicine, Department of Family Medicine, Department of Epidemiology & Biostatistics, Schulich School of Medicine & Dentistry, Western University, 1151 Richmond Street, London, ON, N6A 3K7, Canada, 1 519-661-2111 ext 20049, 1 519-858-5029; 2 Transdisciplinary Understanding and Training on Research-Primary Health Care (TUTOR-PHC) Program, Western University, London, ON, Canada; 3 Graduate Program in Epidemiology & Biostatistics, Department of Epidemiology & Biostatistics, Schulich School of Medicine & Dentistry, Western University, London, ON, Canada; 4 Arthur Labatt Family School of Nursing, Western University, London, ON, Canada

**Keywords:** patient engagement, patient-oriented research, patient and public involvement, primary health care research, primary care research, capacity-building, training, course evaluation, co-design

## Abstract

**Background:**

Patient engagement in research represents an evolution in how new knowledge is being created. Individuals and teams seeking to conduct research in this way want to learn how to best approach this aspect. Specialized training is required to ensure that these individuals and groups have the knowledge and skills to engage with and accomplish these goals. We developed a training program, called Patient-Oriented Research Training & Learning - Primary Health Care (PORTL-PHC), to address this need.

**Objective:**

The objective of this paper was to describe key learning needs and knowledge gaps regarding patient-oriented research in primary health care, as well as the design, implementation, and evaluation of the PORTL-PHC program.

**Methods:**

First, we completed a needs assessment to determine the learning needs of the program’s target groups (including patient partners, policy makers, health care practitioners, and researchers). Second, building on the results of the needs assessment, the development and implementation of the program followed a series of iterative steps, including user testing of the program’s content and format. Third, we conducted an evaluation with two components: (1) program registrants were asked to respond to questions as they progressed through the training content that explored what aspects of the content users found the most useful, suggestions for improvement, and any difficulties navigating the learning platform; and (2) program registrants were administered a questionnaire in three waves (January 2020, July 2020, and September 2021) 6 months after they had completed the program, that asked them to rate their gains in different areas of knowledge and skills regarding patient-oriented research on a 5-point Likert scale.

**Results:**

There were 205 learners who participated in the program from January 2018 to January 2022. The target audience was reached with registrants from all groups; the majority of learners were from Canada (194/205, 95%). A total of 6 main areas of knowledge needs were identified from the needs assessment, and the program was iteratively developed and refined to address these needs and our learning objectives. Suggestions for improvement received from the first component of the evaluation were used to enhance and refine the program. Of the 88 learners who had completed the program at the time of the evaluation questionnaire administration, 28 responded to our request to complete an evaluation. The results indicate that PORTL-PHC increased knowledge of patient-oriented PHC research (overall mean score of 4.36, SD .56). Learners gained skills and knowledge in identifying patient priorities in PHC (mean 4.27, SD .63), understanding the methods of patient engagement (mean 4.32, SD .65), and skills for engagement in patient-oriented research (mean 4.41, SD .50). The majority of respondents (23/28, 82%) indicated that they intended to use the information from the PORTL-PHC training program in the future.

**Conclusions:**

Through the PORTL-PHC program, we are training a new cadre of interested individuals who are committed to patient engagement in research to improve the provision of primary health care, and thus, patient outcomes.

## Introduction

### Background

Patient engagement in research, which has been defined as “The active, meaningful, and collaborative interaction between patients and researchers across all stages of the research process, where research decision making is guided by patients’ contributions as partners, recognizing their specific experiences, values, and expertise” [1], represents an evolution in how new knowledge is being created. This approach respects the fact that patients and the broader public ultimately fund research and thus should be part of its creation and evaluation [2]. As this approach to research has become more widespread, patient partners and researchers have reflected on their experiences [3,4], the impacts of approaching research in this way have been described [5,6], and models and frameworks to guide this work have emerged [7].

Organizations such as the Patient-Centered Outcomes Research Institute [8] in the United States, and the Centre for Engagement and Dissemination at the National Institute for Health and Care Research in the United Kingdom [9], have supported and promoted this work. In 2011, the Canadian Institutes of Health Research (CIHR) launched the Strategy for Patient-Oriented Research (SPOR) [10] and supported SUPPORT Units across Canada to enact the SPOR strategy. The SPOR Patient Engagement Framework states that “Patient-oriented research refers to a continuum of research that engages patients as partners, focuses on patient-identified priorities and improves patient outcomes. This research, conducted by multidisciplinary teams in partnership with relevant stakeholders, aims to apply the knowledge generated to improve healthcare systems and practices” [10]. The goal of SPOR was to engage patients, caregivers, and families as partners in the research to make sure that health research focused on priorities of patients. CIHR developed the SPOR initiative to help transform the role of patients in the research process and to change the way research was being conducted in Canada [10,11]. As a result, there are many patient-oriented health research initiatives that exist [12,13], including the Passerelle program, which is the main hub for patient-oriented research training and capacity building in Canada [14]. Other developments include new patient-led initiatives such as the PxP For Patients, By Patients [15], and centres such as the Patient Expertise in Research Collaboration—Primary Health Care [16]. Please note that, in this paper, we use both the terms patient engagement in research and patient-oriented research.

Individuals and teams (including patient partners, policy makers, health care practitioners, and researchers) seeking to conduct and use patient-oriented research want to learn how to best approach this work. They want to ensure that patients’ voices are heard, make sure that the research produced is relevant to patients, and ultimately to improve the health of patients [17]. Specialized training is required to ensure that these individuals and groups have the knowledge and skills to engage with and accomplish these goals [2]. Beginning in 2014, the Ontario SPOR SUPPORT Unit (OSSU) funded a suite of training and capacity building initiatives to respond to this need for specialized training in patient-oriented research [18-21]. In addition, the OSSU publishes a compendium of patient-oriented research capacity building programs and resources across Ontario, reflecting the evolving and expanding nature of these initiatives [22].

Members of our team are active in developing and delivering research training initiatives focused in the primary health care setting. Therefore, we knew that (1) it was important to provide specialized training so that individuals would know how to engage with and conduct patient-oriented research; and (2) that this training should focus on the primary health care setting and its patients, to best match the perspectives and learning needs of patients, practitioners, policy makers and researchers in this setting, which includes services provided by primary care practitioners. Recognized as the “foundation of the health care system” [23], primary care is characterized by essential attributes known as the 4Cs—“first contact, comprehensiveness, coordination, and continuity” [23,24]. The scope of primary care in terms of the health care system is large—most of the care provided in health care systems in terms of monthly contacts for example occurs in primary care [25]. Therefore, we developed a training program to address the unique needs of learners in the primary health care setting [26]. The program was funded by the OSSU as part of its original suite of capacity building initiatives. The training program is called Patient-Oriented Research Training & Learning-Primary Health Care (PORTL-PHC) and is hosted on The University of Western Ontario’s (UWO) Learning Management Platform called OWL. The goal of PORTL-PHC was to build capacity among patients, health care providers, policy makers or managers, researchers and trainees to conduct and use patient-oriented primary health care research. This work was conducted in two main phases, which involved (1) the collection of foundational information about learning needs and gaps in knowledge regarding primary health care patient-oriented research; and (2) the design, delivery, and evaluation of the program.

This paper reports on the key learning needs and knowledge gaps that were identified, as well as the design, implementation, and evaluation of the PORTL-PHC program.

### Principles Underpinning the Creation and Design of PORTL-PHC

The overarching principles that underpinned the creation of the program were to ensure that co-design and co-building processes were used from the start of the original program proposal to the final development and delivery of the program; the training program would meet the needs of multiple interested groups, the perspectives of potential end-users were incorporated throughout the process, and the content would reflect the primary health care research context.

In keeping with these principles, we struck an Advisory Committee with representatives from four groups (patients, primary health care practitioners, policy makers, and researchers). The committee provided input, feedback, and guidance for the main activities of the program, including curriculum design, content and delivery, engagement strategies and recruitment, and evaluation, as well as identifying appropriate resources to support the project over the short and long term.

The project team closely followed the overarching principles throughout the program development process. Representing the patient perspective, co-authors (LB and LM) were engaged at the beginning stage of the proposal development for the project and were an integral part of the development and user testing of the program. LB and LM supported the creation of the program by: (1) attending all PORTL-PHC team meetings, (2) identifying new materials for the program, (3) contributing to logic model and evaluation design, (4) reviewing materials, (5) testing the program, and (6) making connections to promote the program within their own networks. They engaged a significant number of patients, caregivers, and citizens to provide input at the needs assessment stage of the project. An additional patient partner was a member of the Advisory Committee.

## Methods

### Learning Needs and Knowledge Gaps: Data Collection and Analysis

To ensure that the program addressed existing knowledge gaps regarding patient-oriented research, we completed a needs assessment in 2 main steps to determine the learning needs of the targeted groups. First, we conducted a review of relevant documents regarding the learning needs of these groups, including reports prepared for the OSSU’s MasterClass on Patient-Oriented Research [27], and the Canadian Institutes of Health Research’s Evaluation of the Strategy for Patient-Oriented Research [28]. A total of 2 study authors (ALT and RVH) collated this information and categorized it into broad thematic areas.

Second, we conducted an informal survey to explore learning needs for participating in, conducting, or using patient-oriented primary health care research. We developed a short questionnaire based on a brief review of literature and the document review described above. The questionnaire was designed to elicit responses regarding interest in participating in patient-oriented research, what type of knowledge and learning individuals were looking for in a training program, what topics were most important to address, and whether they had ever participated in patient-oriented research previously. Research team members and members of the Advisory Committee iteratively reviewed the questionnaire to improve clarity and to adjust the content. The questionnaire was administered through Qualtrics, which is an survey software program [29]. Qualtrics was used for the remainder of the data collection activities described in this methods section. Networks and programs relevant to primary health care and patient- oriented research across Ontario, Canada were asked to distribute the questionnaire to their members. Descriptive statistics were calculated to summarize the quantitative data. A total of 2 study authors (ALT and RVH) reviewed and summarized responses to the open-ended questionnaire elements.

### PORTL-PHC Program Design

Building on the results of the needs assessment, the development and implementation of the program followed a series of iterative steps. First, we developed educational objectives that served as a guide for the content of the program. Second, using the information gathered in the learning needs assessment, we developed the structure and content of the program. The overall design was guided by adult learning principles [30] using tested pedagogic and andragogic approaches for both content and process. Approaches include research skills development [31], explicit knowledge [32], tacit knowledge [32], collaborative co-created learning [32], critical reflection [33], educating for capability [34], and building a community of scholars. Building on Knowles’ [30] “self-concept” principle, we set out to design the program to allow the learner to individualize their experience by exploring the content in a way that would be most helpful to them and pertinent to their immediate needs. Third, the content and structure of the program were configured for self-directed learning within the learning platform. Aspects of the visual display, site navigation, and structure were created and refined, and then, the content was added. Fourth, after the initial version of the training program was developed, we conducted a series of steps in user testing and program refinement. PORTL-PHC Advisory Committee members reviewed and tested the program; their feedback on the appearance, structure, and content of the modules and the overall design was incorporated into a revised version of PORTL-PHC. Partner organizations of the PORTL-PHC program including the Patient Expertise in Research Collaboration (PERC), the Centre for Rural and Northern Health Research (CRaNHR) and Innovations Strengthening Primary Healthcare through Research–Primary Health Care (INSPIRE-PHC) were then asked to provide names of potential program user testers associated with their organizations. These user testers—5 patients, 2 researchers, 1 policy maker, and 1 research trainee—were asked to complete the program, provide feedback on the content, and assess the site’s functionality, the appearance of the program, the design, and the clarity of the instructions. The input received was used to revise the appearance, content, and design of the PORTL-PHC training modules and website.

### PORTL-PHC Program Recruitment and Promotion

A variety of methods were used to promote the program including information circulated to: the OSSU; SUPPORT Units and Primary and Integrated Health Care Innovations Networks (PIHCINs) in each province across the country; OSSU Member Centers including INSPIRE-PHC and CRaNHR; Patient Expertise in Research Collaboration (PERC); Transdisciplinary Understanding and Training on Research—Primary Health Care (TUTOR-PHC) alumni network; patient networks such as the Patient Advisory Network (PAN); mailing lists of these connected networks, newsletters such as in the Department of Family Medicine at Western University and on social media via X (formerly known as Twitter). We also promoted the program, and shared early findings about its implementation and uptake, by making presentations about PORTL-PHC at primary health care Research Conferences such as the North American Primary Care Research Group Annual Meeting [35] and the Trillium Primary Health Care Research Day [36,37], as well as advertising with bookmarks and brochures available to conference attendees.

### PORTL-PHC Program Evaluation

**Figure 1.**
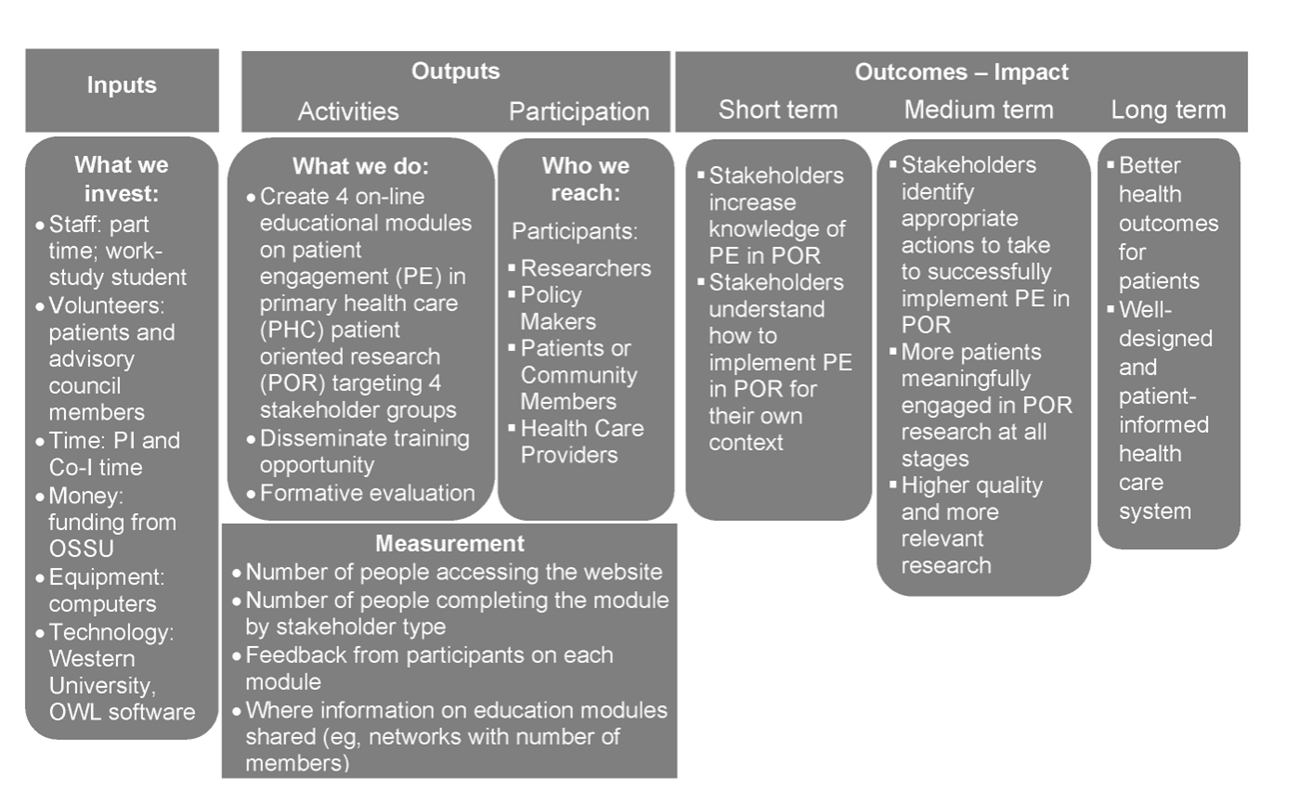
Program logic model. Co-I, co-investigator; OSSU, Ontario SPOR SUPPORT Unit; OWL, Western University's online learning management system; PI, principal investigator; UWO, The University of Western Ontario.

The overall evaluation of the program was informed by Kirkpatrick’s 4-level training evaluation model [38] and guided by a logic model developed for this purpose (see Figure 1); we measured outputs and assessed short-term impacts in this phase of the project. Data collection for evaluation purposes occurred in four ways. First, learners were asked to provide their group and location upon registration. Second, we administered a questionnaire to new learners in the program, requesting information about their experience participating in or using patient-oriented research, how they identified the training program, and their affiliation with any patient-oriented research organizations. Third, learners were asked to complete a series of questions at the end of each module that explored what aspects of the module users found the most useful, suggestions for improvement, and any difficulties navigating the learning platform; this information was collected through a questionnaire embedded at the end of each module. Finally, we conducted a survey of learners in three waves (January 2020, July 2020, and September 2021) 6 months after they completed the program to ascertain if the learning objectives for the training program were met. One follow-up reminder was sent to learners who had not completed the evaluation questionnaire. We also collected information on where the learners were located, and category of learner (ie, administrative staff [eg, project coordinator, research assistant]), patient or caregiver, student or trainee, primary health care researcher, health care practitioner, and policymaker or manager. We calculated descriptive statistics to summarize these data.

### Ethical Considerations

For the survey component of the needs assessment (described in the “Learning Needs and Knowledge Gaps: Data Collection and Analysis” section above), participants reviewed a letter of information before consenting to participate. No personal identifiers were collected and no compensation was offered for participation. This project was approved by the UWO Health Sciences Research Ethics Board (109621). Additional activities (described in the “Program Evaluation” section above) are program evaluation activities and therefore would be considered exempt from human ethics review in accordance with Article 2.5 of the Tri-Council Policy Statement: Ethical Conduct for Research Involving Humans, which states that “Quality assurance and quality improvement studies, program evaluation activities, and performance reviews, or testing within normal educational requirements when used exclusively for assessment, management or improvement purposes, do not constitute research for the purposes of this Policy, and do not fall within the scope of REB review” [39].

## Results

In this section, we present the results of the steps undertaken in our needs assessment (see “Learning Needs and Knowledge Gaps: Results” section), followed by the results of our program design process (see “PORTL-PHC Program Design: Results” section), and finally, the process and outcome results of the PORTL-PHC program evaluation (see “PORTL-PHC Program Evaluation: Process and Outcome Results” section).

### Learning Needs and Knowledge Gaps: Results

In the first step of our needs assessment, overall themes emerged from the document review we conducted regarding learning needs and knowledge gaps for patient-oriented-research, as well as those that related to specific groups; Textbox 1 shows these themes.

Textbox 1. **Document review results**

Overall themes included a need for:

- Basics of patient-oriented research, definitions, frameworks, and methods.
- Concrete information or steps regarding conducting patient-oriented research, tools, skills development, and understanding enablers and barriers.
- Information regarding ethics and patient-oriented research.
- Examples of patient-oriented research and “learning by doing” exercises and simulations.
- Clear articulation of roles of members of the research team, for example, co-building.

Groups and their themes:

Patients:

- Ensuring patient perspectives are included and valued.
- Need for technical research knowledge—curriculum vitae, ethics, report writing, and granting processes.
- Issues in conflicting priorities among different groups and organizations.
- How to engage in patient-oriented research?
- Role on research teams—need for clarity, participation at the right time.
- Knowledge regarding existing research and how it can be applied.

Practitioners:

- Assessing patient needs or balancing priorities.
- Need for resources (funding and literature).
- Identifying and engaging patients and partnerships.

Policy makers:

- Access to relevant information.
- Culture change required regarding value of patient engagement.
- Need for resources to support patient-oriented research and capacity for patient engagement.
- Tension regarding the need for representative evidence versus qualitative information.
- Time and resources.

Researchers:

- Finding or accessing patient members.
- Understanding the best way to include patients in research and the right type of involvement for each project.
- How to elicit, incorporate, or balance patient priorities and preferences?
- How to handle language and terminology differences?
- Understanding and demonstrating the value of patient engagement in research.
- Need for evaluation and outcome measures to assess patient engagement and its impact.
- What are the long-term strategies and vision for patient-oriented research?

**Figure 2.**
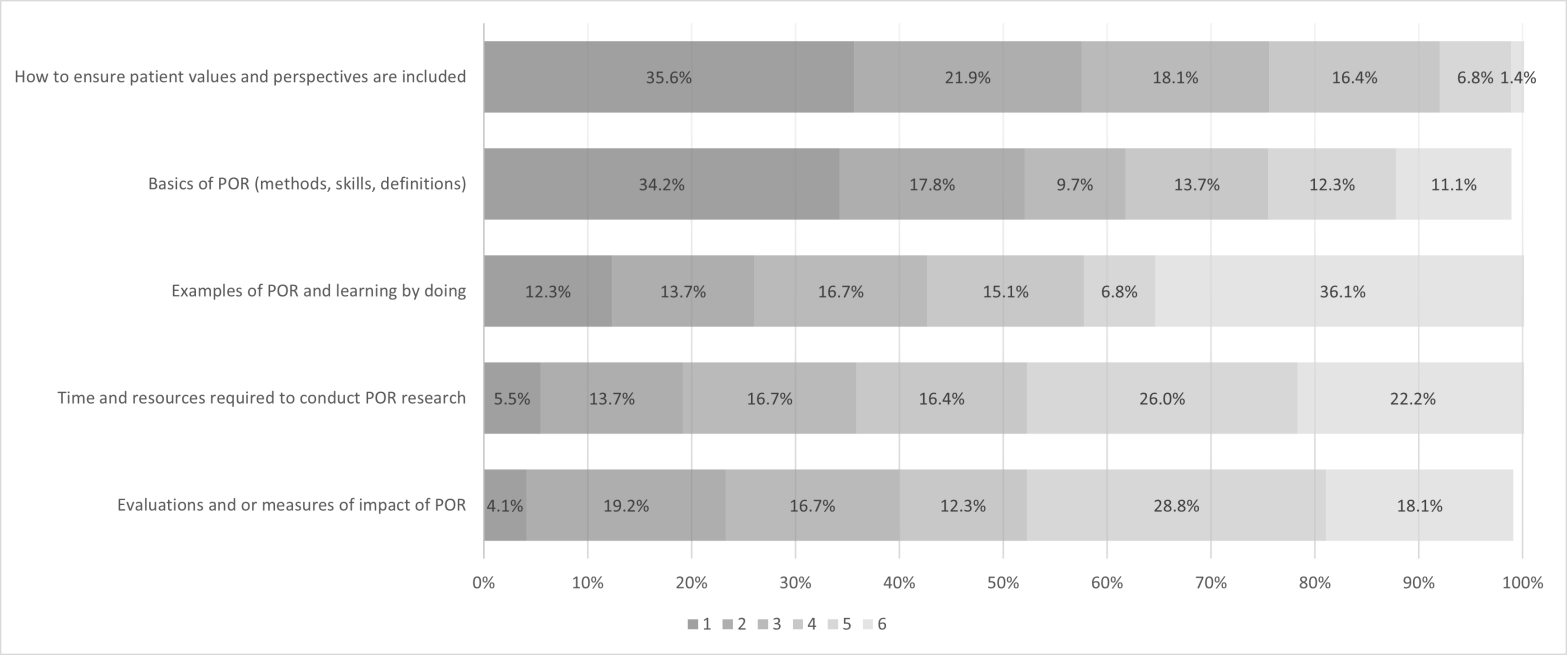
Ranking of topics for inclusion in a patient-oriented research (POR) training program (N=73). Participants were asked to rank the listed topic from 1 to 6, with 1 being most important.

For the second step of the needs assessment, 75 individuals responded to the PORTL-PHC learning needs assessment questionnaire. Most respondents were primary health care researchers (31/75, 41%) or patients (17/75, 23%), followed by students or trainees (9/75, 12%), clinicians (6/75, 8%), with the remainder being caregivers, other, or policy makers or managers (12/75, 16%). The majority of respondents (66/75, 88%) expressed interest in participating in a patient-oriented research training program, with just over half (39/75, 52%) having ever participated in, or previously used, patient-oriented research. Of the 73 respondents who answered questions about topic preferences, the basics of patient-oriented research and ensuring the inclusion of patient values and perspectives were consistently the highest ranked topics for inclusion in a patient-oriented research training program, while other topics such as roles on research teams, time and resources required to conduct patient-oriented research, and evaluating the impact of patient-oriented research were of lower priority (see Figure 2).

In total, 6 main areas of knowledge needs were identified through a synthesis of the open-ended survey questions. Respondents were seeking information about the “basics” of patient-oriented research, such as how to recruit patients. They wanted an understanding of the roles that patients take on in research, and how to ensure that patient values and perspectives were included. Information regarding the time and resources required to conduct patient-oriented research was important. Respondents were seeking examples of patient-oriented research, best practices, and lessons learned. Finally, they wanted to know how to evaluate their patient-oriented research work and understand its impact.

### PORTL-PHC Program Design: Results

The results of our program design steps included the development of five cross-cutting educational objectives of the PORTL-PHC program, which are as follows: (1) to develop an understanding of the experiences of primary health care patients; (2) to gain knowledge of approaches to identifying patient priorities in primary health care; (3) to understand methods of how to engage and be engaged in patient-oriented research, and how to listen to patient voices; (4) to develop knowledge and skills in conducting and participating in patient-oriented research, in using patient-oriented research, and in an outlook that supports effective patient engagement; and (5) to actively apply patient-oriented research skills and knowledge in the learners’ own context. A total of five learning modules, described in Table 1, were created to address these educational objectives. The design and delivery methods for each module include seven common components (see Table 2).

**Table 1.** Overview of Patient-Oriented Research Training & Learning—Primary Health Care (PORTL-PHC) program: module objectives and description.

Module	Educational objectives addressed	Description
Module 1A and 1B	First, to develop an understanding of the experiences of primary health care patients. Second, to gain knowledge of approaches to identifying patient priorities in primary health care.	Patient priorities and patient engagement in primary health care research: Module 1A focuses on learning what the “big picture” issues are for primary health care patients. It provides information to all interested groups about what is important to primary health care patients in terms of their needs and priorities. Module 1 B provides information about how to identify patient priorities for primary health care research. This module discusses some of the methods for involving patients in identifying priorities for research and provides some real-world examples.
Module 2	Third, to understand methods of how to engage and be engaged in patient-oriented research, and how to listen to patient voices.	Methods and examples of patient engagement in primary health care research: Module 2 focuses on approaches to engage patients in research. Methods which go along with each level of patient engagement are illustrated though examples of real-world studies. Relevant content addresses how to listen to patient voices throughout each of the levels or stages of patient engagement in research.
Module 3	Fourth, to develop knowledge and skills in conducting and participating in patient-oriented research, in using patient-oriented research, and in an outlook that supports effective patient engagement.	Skills development in patient engagement and patient-oriented research: Module 3 focuses on the knowledge, skills, and outlook needed to participate in patient-oriented research, to conduct patient-oriented research, or to use this type of research. The module aims to identify gaps in knowledge, skills, and outlook for learners. After identifying these gaps, learners are directed to seek out the necessary resources and examples presented in the program modules to address these gaps.
Module 4	Fifth, to actively apply patient-oriented research skills and knowledge in the learners’ own context.	Applying patient-oriented research in the learner’s own context: Module 4 focuses on applying the learnings from Modules 1 through 3 to the learner’s own perspective as a patient, or work as a researcher, policy-maker, or health care practitioner. Based on each learner’s perspective, this module focuses on real-world application of ways to be involved in patient-engaged research, opportunities and challenges, and means to evaluate these projects.

**Table 2.** Overview of Patient-Oriented Research Training & Learning—Primary Health Care (PORTL-PHC) program: the 7 common components of the delivery methods and designs of each module.

Component	Description
Introduction	An overview of the topic, explanation of how to use the training, why the training was created, and what learners could expect from the training.
Content	Slides, video (including patient perspectives), and text were used to deliver relevant content. Using different types of media allowed learners with different learning styles (visual, auditory, and kinesthetic) to maximize their learning experience.
Existing resources	Links to existing resources for all sections of the modules.
Examples	Experiences of team or advisory group members and actual POR^a^ work were used as examples.
Exercises	Dynamic exercises that include built in questions leading to different content for different learner groups.
Self-reflection	Self-reflection questions or short quizzes based on content.
Feedback	Feedback opportunities via evaluation questions.

a POR: patient-oriented research.

Thus, for each module, learners were able to review pertinent content regarding primary health care patient-oriented research, work through a series of examples and exercises, engage in self-reflection, and provide feedback. This feedback was reviewed with a view to further enhancing the program. Within an e-learning environment, the program guides the learner and provides ample resources while allowing them to “discover” much of the information and incorporate it as needed [40]. This is a self-directed program, where learners can move through the modules at their own pace, according to their schedules. Each learner is registered individually to the learning platform and has unlimited access to the program’s content.

The final version of the program was created and launched via OWL (UWO’s Online Learning Management System) in December 2018. Ongoing support for the OWL platform through UWO allows the PORTL-PHC program to be sustained over time. A comprehensive review of the program’s content and resources was conducted in 2023; updated materials and links to new resources were added to the program site.

### PORTL-PHC Program Evaluation: Process and Outcome Results

There were 205 learners who participated in the program from January 2018 to January 2022 (see Table 3). The target audience was reached with registrants from all target groups; the majority of learners were from Canada (194/205, 95%). Of the 133 registrants who responded to a question about their patient-oriented research experience, more than half (68/133, 51%) had participated in or used this type of research. Responses to questions posed at the end of each module about the aspects of the module that were most useful, suggestions for improvement, and any challenges in navigating the website indicate that that the content and delivery platform was well-received by learners. Suggestions for improvement were used to enhance and refine the program.

**Table 3.** Profile of Patient-Oriented Research Training & Learning—Primary Health Care (PORTL-PHC) program learners (N=205).

Characteristics	Values, n (%)
Country of Residence	
Canada	194 (94.6)
United States	6 (2.9)
Other (Australia, Japan, Pakistan, and Qatar)	5 (2.4)
Learner category	
Administrative staff (eg, Project coordinator, research assistant)	59 (28.8)
Patient or caregiver	40 (19.5)
Student or trainee	36 (17.6)
Primary health care researcher	29 (14.1)
Health care practitioner	28 (13.7)
Policymaker or manager	13 (6.3)

We conducted an evaluation survey in 2020-21 with learners who fulfilled two criteria: (1) they had completed the PORTL-PHC program; and (2) they had completed the program at least 6 months before the survey time period. This meant there were a total of 88 learners eligible to participate. On administration of the evaluation questionnaire, 34 individuals began to complete the questionnaire, and 28 individuals finished (32% response rate; see Table S1 in Multimedia Appendix 1). The vast majority of the respondents were from Canada; two-thirds of the group was made up of researchers and administrators with the remainder a mix of clinicians, trainees, and patients or caregivers. Respondents indicated that the PORTL-PHC training program had increased their knowledge of patient-oriented primary health care research (overall mean score of 4.36, SD .56, five response options from strongly disagree to strongly agree were scored 1 through 5). Learners gained skills and knowledge in areas such as identifying patient priorities in primary health care (mean 4.27, SD .63), understanding the methods of patient engagement (mean 4.32, SD .65), and skills for engagement in patient-oriented research (mean 4.41, SD .50). The majority of respondents (23/28, 82%) indicated that they intended to use the information from the PORTL-PHC training program in the future. Respondents were also asked several open-ended questions about how the PORTL-PHC training program helped shaped their research goals and to explain how knowledge gained from the program was used to shape and design their research initiatives. Respondents indicated that they applied the learnings from the program in a variety of ways, such as using the training to develop their own research methods, to conducting peer reviews, and to critique patient engagement in research projects. Respondents noted that the program provided clarification about what was involved in patient-oriented research and gave the learners confidence in joining research teams or implement patient-oriented research-related activities.

## Discussion

### Principal Findings

In building the PORTL-PHC program, we used an iterative and collaborative process to ensure that our principles of co-design and co-development that supported the creation and delivery of the program were upheld. These principles included having patient partners, practitioners, policy makers, and researchers involved from the start of the program development to its final delivery, designing a program to meet the needs of multiple groups, capturing and addressing the perspectives of end users, and ensuring that the content of the program was highly relevant to the primary health care context. The experience of co-designing and developing the PORTL-PHC program further heightened our shared awareness of the value of end-users shaping the program to meet their needs. Iteratively seeking input on the program allowed us to capture feedback provided by all interested groups, including patients, and refine the program accordingly. This resulted in a highly relevant program that has been successfully taken up by learners in Canada and internationally. We plan to apply this model of assessing needs, co-design, and iterative refinement in our future research and educational program development initiatives.

The main areas of knowledge needs identified in our needs assessment process included basic knowledge of methods and skills in patient-oriented research, understanding patients’ roles in research, ensuring patient values and perspectives were included, understanding the time and resources required to conduct patient-oriented research, having exemplars of research and best practices, and how to evaluate or measure the impact of patient-oriented research. These areas of knowledge needs formed the basis of the program’s content. Following an iterative design process, we developed cross-cutting educational objectives for the program and created 5 learning modules to address these objectives. The PORTL-PHC program includes modules that lead the learner through a series of topics regarding patient experiences in primary health care, identifying patient priorities in primary health care, methods of how to engage and be engaged in patient-oriented research, development of knowledge and skills around patient engagement in research, and how to apply the knowledge gained in the learner’s own context. Responses to questions posed to each learner about the module content and format were used to enhance the overall program. Evaluation results indicate that the program met its educational objectives, with learners indicating that they had increased their knowledge and skills in patient-oriented research, and that they would use the information from the program in their future work. The results also suggest that the program was responsive to user needs, reached the target audience, and heightened the awareness and knowledge of multiple groups including patients, policy makers, practitioners, and researchers.

As patient and community engagement in research continues to grow and mature, it will be increasingly important to have a suite of options available for interested individuals to participate in training to enhance their knowledge and skills in co-creating patient-oriented research. The possibility of coordinated offerings of such training programs as outlined by Chudyk et al [41] represents an ideal to strive toward. Initiatives such as Canada’s Passerelle Program are important developments that support this aim; the Passerelle program is a national training entity and a central pan-Canadian hub that brings together networks and programs to support capacity development in patient-oriented research [14]. PORTL-PHC is actively collaborating with Passerelle around the shared goal of providing enhanced patient-oriented research training in Canada. PORTL-PHC is a sustainable program that is designed to facilitate capacity building and strengthen efforts to engage patients as partners in primary health care research. By providing primary health care specific exercises, examples and resources, we addressed the needs of our learners by attending to the unique context within which primary health care research occurs. Part of the success of the program lies in the foundational work conducted to understand the knowledge needs of our learners, the engagement of the target audiences in our design process, and the testing and subsequent refinement of the program with interested individuals and groups. Our training program was developed at a stage when patient engagement in research was earlier in its emergence, yet there is an ongoing demand for the PORTL-PHC program itself, and an overall need for this type of training to carry on [2]. Although guidance regarding patient-engagement in research continues to emerge [42], the PORTL-PHC program responds to a specific need by delivering training tailored to the primary health care setting; addressing a gap in current educational offerings focused on engaging patients in research.

### Strengths and Limitations

Several strengths of the PORTL-PHC program include: (1) the extent of the engagement with patients and other partners in its development, (2) the responsiveness to the findings of our needs assessment in creating the program’s content, and (3) the iterative nature of user testing and development of the program. The evaluation results indicate that the PORTL-PHC program is achieving its objectives and attracting its target audience. The self-directed nature of the program allows us to sustain the program’s delivery and the openly accessible learning platform means that we can provide the program to all who are interested [43]. Several limitations must be noted, and include: (1) the fact that the evaluation results are based on self-reported data from approximately a third of participants, (2) there is an overrepresentation of primary health researchers and an underrepresentation of health care practitioners and policy makers in the evaluation survey respondent group, (3) the program is offered in OWL and therefore assumes access to a computer and internet connectivity, and (4) and that the program is currently only offered in English.

### Conclusions

Through the PORTL-PHC program, we are training a new cadre of interested individuals who are committed to patient engagement in research to improve the provision of primary health care, and thus, patient outcomes. In particular, primary health care researchers and health care practitioners are able to partner with patients in a meaningful way in their research, and patients and policy makers are better prepared for participation in primary health care research.

## Supplementary material

10.2196/65485 Multimedia Appendix 1 Patient-Oriented Research Training & Learning—Primary Health Care (PORTL-PHC) evaluation questionnaire responses (n=28).

## Abbreviations

CIHR: Canadian Institutes of Health Research
CRaNHR: Centre for Rural and Northern Health Research
INSPIRE-PHC: Innovations Strengthening Primary Healthcare through Research
NIHR: National Institute for Health and Care Research
OSSU: Ontario SPOR SUPPORT Unit
OWL: Western University's online learning management system
PAN: Patient Advisory Network
PERC: Patient Expertise in Research Collaboration
PHC: primary health care
PIHCINs: Primary and Integrated Health Care Innovations Networks
PORTL-PHC: Patient-Oriented Research Training & Learning - Primary Health Care
SPOR: Strategy for Patient-Oriented Research
TUTOR-PHC: Transdisciplinary Understanding and Training on Research—Primary Health Care
UWO: The University of Western Ontario
