# Supplementary material for: A Training Program to Support Patient Engagement in Primary Health Care Research: Co-Design, Implementation, and Evaluation Study
Source: J Particip Med. 2025 Jun 5;17:e65485. doi: 10.2196/65485 (PMC12161618; doi:10.2196/65485)
Supplement: Multimedia Appendix 1 [file jopm-v17-e65485-s001.docx]

**Multimedia Appendix 1**

**Table- PORTL-PHC Evaluation Questionnaire Responses (n=28)**

|  | N | Mean (SD) | Med | Min | Max |
| --- | --- | --- | --- | --- | --- |
|  |  |  |  |  |  |
| **Question** |  |  |  |  |  |
|  |  |  |  |  |  |
| After taking the PORTL-PHC training program, my knowledge of Patient-Oriented Research has increased | 28 | 4.36 (0.56) | 4 | 3 | 5 |
|  |  |  |  |  |  |
| Have you gained the following Patient Oriented Research skills/ knowledge since taking the PORTL-PHC training program? |  |  |  |  |  |
|  |  |  |  |  |  |
| a) Developed an understanding of the major topics and issues experienced by patients in primary health care | 22 | 4.18 (0.66) | 4 | 3 | 5 |
|  |  |  |  |  |  |
| b) Identified approaches to identifying patient priorities in primary health care | 22 | 4.27 (0.63) | 4 | 3 | 5 |
|  |  |  |  |  |  |
| c) Understood methods of how to engage and be engaged in Patient-Oriented Research | 22 | 4.32 (0.65) | 4 | 3 | 5 |
|  |  |  |  |  |  |
| d) Learned how to listen to patient voices | 22 | 4.32 (0.72) | 4 | 3 | 5 |
|  |  |  |  |  |  |
| e) Gained skills for engagement in patient-oriented research | 22 | 4.41 (0.50) | 4 | 4 | 5 |
|  |  |  |  |  |  |
| f) Ability to apply patient-oriented research in own setting | 22 | 4.18 (0.73) | 4 | 3 | 5 |
| **Response options on a 5-point scale were: Strongly Disagree, Disagree, Neutral, Agree, Strongly Agree)** | | | | | |

N=number of respondents, SD=standard deviation, Med=median, Min=minimum, Max=maximum
